# Concept and design of a nationwide prospective feasibility/efficacy/safety study of weekly paclitaxel for patients with pathologically confirmed anaplastic thyroid cancer (ATCCJ-PTX-P2)

**DOI:** 10.1186/s12885-015-1490-8

**Published:** 2015-06-20

**Authors:** Naoyoshi Onoda, Iwao Sugitani, Takuya Higashiyama, Hisato Hara, Ken-ichi Ito, Makoto Kammori, Kiminori Sugino, Shinichi Suzuki, Kazuhisa Toda, Akira Yoshida, Akira Miyauchi

**Affiliations:** 1 Prospective Clinical Study Committee of Anaplastic Thyroid Carcinoma Research Consortium of Japan, Headquarter in the Department of Endocrine Surgery, Nippon Medical School, 1-1-5 Sendagi, Bunkyo-ku, Tokyo, 113-8603 Japan; 2 Department of Surgical Oncology, Osaka City University Graduate School of Medicine, 1-4-3 Asahi-machi, Abeno-ku, Osaka, 545-8585 Japan; 3 Department of Endocrine Surgery, Nippon Medical School, 1-1-5 Sendagi, Bunkyo-ku, Tokyo, 113-8603 Japan; 4 Kuma Hospital, 8-2-35 Shimoyamate-dori, Chuo-ku, Kobe-shi, Hyogo 650-0011 Japan; 5 Department of Breast and Endocrine Surgery, Tsukuba University, 1-1-1 Tenno-dai, Tsukuba-shi, Ibaragi 305-0006 Japan; 6 Department of Surgery II, Shinshu University, 3-1-1 Asahi, Matsumoto-shi, Nagano, 390-8621 Japan; 7 Kanaji Hospital, 1-5-6 Nakazato, Kita-ku, Tokyo, 114-0015 Japan; 8 Ito Hospital, 4-3-6 Jingu-mae, Shibuya-ku, Tokyo, 150-8308 Japan; 9 Department of Thyroid and Endocrinology, Fukushima Medical University School of Medicine, 1 Hikarigaoka, Fukushima, Fukushima 960-1295 Japan; 10 Division of Head and Neck, Cancer Institute Hospital, 3-8-31 Ariake, Koto-ku, Tokyo, 135-8550 Japan; 11 Department of Breast and Endocrine Surgery, Kanagawa Cancer Center, 2-3-2 Nakao, Asahi-ku, Yokohama-shi, Kanagawa 241-8515 Japan

**Keywords:** Anaplastic thyroid cancer, Chemotherapy, Prospective study, Feasibility, Protocol, Paclitaxel

## Abstract

**Background:**

Anaplastic thyroid cancer (ATC) is one of the most aggressive malignancies in humans, often demonstrating resistance to multimodal therapeutic approaches. The median survival of ATC patients after initial diagnosis was reported to be < 6 months due to the rapid progression of disease by dissemination and/or invasion. There have been several reports describing possible effective chemotherapies, but these studies might be biased by the nature of retrospective accumulations of clinical experiences, and thus reliable data concerning the efficacies of the treatment efforts are required.

**Design:**

In 2009, we established the research organization Anaplastic Carcinoma Research Consortium Japan (ATCCJ) to investigate this highly malignant disease. Using this nationwide organization, we conducted a prospective clinical study to investigate the feasibility, safeness, and efficacy of chemotherapy with weekly paclitaxel for ATC patients. This trial is registered on the clinical trials site of the University Hospital Medical Information Network Clinical Trials Registry Web site (UMIN000008574). The study was started in 2012, and enrollment was closed in March 2014 after accumulating 71 patients from 28 registered institutes. The follow-up data will be available in April 2015.

**Discussion:**

Important information concerning the management of this disease is expected to be revealed by this study. The concept and design of the study are described herein.

## Background

The management of anaplastic thyroid cancer (ATC) is often difficult because of its aggressive characteristics and the acquisition of resistance to multimodal therapeutic approaches [1]. The rareness of ATC (it accounts for 1 % –4 % of thyroid cancer cases) has resulted in a lack of accurate information concerning the disease, including the outcomes of treatment efforts [2]. Long-term experiences from single institutes may suggest different strategies to manage this disease [3, 4], and an analysis using a common database lacked information concerning the treatment efforts [5].

We therefore established the Anaplastic Thyroid Carcinoma Research Consortium of Japan (ATCCJ) in 2009 to accumulate the clinical data of ATC patients throughout Japan and to construct an authentic large database [6]. Precise data (including the clinical manifestations of the patients, the therapeutic methods used, and the outcomes) were accumulated in January 2010 and are updated annually to form the world’s largest database of ATC cases — over 1,000 cases from 57 institutions as of January 2014 [7]. In an analysis of the ATCCJ data, the 6-month disease-specific survival rates of the ATC patients in the database with stage IVA, B, and C disease were revealed to be 60 %, 45 %, and 19 %, respectively. Age over 70 years, acute symptoms, leukocytosis, tumor dia. > 5 cm, extrathyroidal invasion, and distant metastasis were revealed to be significant poor-prognosis factors [8]. Survival after surgery was significantly better when curative resection was accomplished, or when the disease was observed incidentally during pathological examination after surgery [9]. Surgery, extra-beam radiotherapy and/or chemotherapy may contribute to longer survival in some ATC cases [8].

These findings are important in the choice and creation of therapeutic strategies. However, the practical methods of treatment for ATC are not yet settled, because of the nature of the analyses using retrospective data accumulation.

Conventional chemotherapeutic treatment for ATC using doxorubicin or cisplatinum did not demonstrate a significant effect. The results of several investigations suggested that taxane might be a good candidate agent for controlling the disease [2, 10–13]. Higashiyama et al. reported that the weekly administration of paclitaxel could be beneficial to extend the survival of patients with stage IVB ATC, without causing severe adverse effects [12]. However, there are no precise data regarding which ATC patients could safely undergo chemotherapy, or about the objective response rate to the chemotherapy. The present nationwide prospective clinical study to evaluate the feasibility of weekly paclitaxel administration was conducted with ATC patients at every stage and conditions with the goal of determining the feasibility, safety, and efficacy of this protocol. The study entrée ended on March 2014, and the data will be available in April 2015.

## Methods and design

### Objective

This study was conducted to prospectively evaluate the feasibility and the safety of weekly paclitaxel chemotherapy for patients with ATC. The efficacy of the treatment was evaluated in patients who had evaluable lesions.

### Primary endpoints

For the evaluation of the chemotherapy’s feasibility and safety, we assessed the relative dose intensity and the occurrence of adverse events. The study committee estimated that approx. 80 % of the intended-to-treat patients would complete the initial course treatment with the intended dose. We also evaluated the overall response rate, time to failure, type of recurrence, and overall survival in the patients with evaluable lesions.

### Study design

The study was a multicenter, nonrandomized, open-label, single-arm Phase II study.

### Eligibility

#### Inclusion criteria

Patients with a pathologically proven diagnosis of anaplastic thyroid cancer aged ≥20 years were eligible for the study if they met the following criteria: Eastern Cooperative Oncology Group (ECOG) performance status of 0 to 2, and adequate bone marrow, hepatic, and renal function. The existence of a Response Evaluation Criteria in Solid Tumors (RECIST) [14]-defined target lesion was not necessary for entry.

#### Exclusion criteria

Major exclusion criteria included previous chemotherapy or radiation therapy for the present illness; suspicion of interstitial pneumonia or pulmonary fibrosis by chest radiograph; brain metastasis with one or more symptoms; the presence of an active peptic ulcer; the presence of another active malignancy; history of severe drug allergy; history of hypersensitivity to polyoxyethylated or hydrogenated castor oil; hypersensitivity to alcohol.

### Ethics and IRB approval

The study protocol was initially approved by the Institutional Review Board (IRB) of the Osaka City University Medical School in March 2012 (#2248). The study was then approved by the IRB at each of the participating institutions (Table 1), and was performed in accord with the Declaration of Helsinki and Good Clinical Practice Guidelines. Written informed consent was obtained from all patients before study entry. This trial is registered on the clinical trials website of the University Hospital Medical Information Network Clinical Trials Registry (UMIN000008574).

**Table 1:** The registered institutions

Institution
1. Osaka City University Hospital, Osaka
2. Kuma Hospital, Kobe
3. Kanaji Hospital, Tokyo
4. Ito Hospital, Tokyo
5. Shinshu University Hospital, Nagano
6. Cancer Institute Hospital, Tokyo
7. Nagasaki University Hospital, Nagasaki
8. Kanagawa Cancer Center, Kanagawa
9. Tsukuba University Hospital, Ibaraki
10. Fukushima Medical University Hospital, Fukushima
11. Nagasaki Medical Center Hospital, Nagasaki
12. Tohoku University Hospital, Sendai
13. Noguchi Hospital, Beppu
14. Aichi Cancer Center Hospital, Nagoya
15. International University of Health and Welfare, Mita Hospital, Tokyo
16. Ida Municipal Hospital, Nagano
17. Mito National Medical Center, Ibaraki
18. Sumitomo Hospital, Osaka
19. Tokyo Medical University Hachioji Medical Center, Tokyo
20. Narita Red Cross Hospital, Chiba
21. National Cancer Center Hospital, Tokyo
22. Uchimaru Hospital, Morioka
23. Iwate Medical University Hospital, Morioka
24. Yokohama City University Hospital, Kanagawa
25. Yokohama City University Medical Center, Kanagawa
26. Kanazawa University Hospital, Ishikawa
27. Nippon Medical School Hospital, Tokyo
28. Kanazawa Medical University Hospital, Ishikawa

### Study protocol

The enrolled patients received intravenous chemotherapy with weekly paclitaxel (80 mg/m^2^, once every week). One course consisted of three administrations. At least one course of the therapy was necessary. Therapy was continued when it was available and the patient’s physician considered it to be appropriate. The dose was reduced to 30 mg/m^2^ when concomitant extra-beam radiation was conducted.

Due to the highly aggressive nature of ATC, a delay in initiating the treatment may result in a critical deterioration of the patient. Therefore, after obtaining the approval of the protocol by each IRB, we asked for institutional registration before each patient’s initial appearance and preparation for acceptance. The protocol treatment was then allowed to be initiated as soon as the patient’s physician had obtained the patient’s consent for entry, upon the pathological diagnosis at the institution. The final pathological diagnosis of ATC was confirmed by a central review by pathologists specializing in thyroid tumors, before the data analysis.

### Assessment

Physical examinations and laboratory tests were performed at baseline and repeated every week. Tumor assessments were performed at baseline and every 3 weeks using the RECIST criteria. Response (complete response/partial response) had to be confirmed more than 3 weeks after it was first noted. Adverse events were reported and graded according to CTCAE v4.0 [National Cancer Institute 2009 Common Terminology Criteria for Adverse Events, version 4.0. Bethesda, MD; U.S. National Cancer Institute].

### Statistical analysis of the responses to therapy

In the present study, the sample size was estimated as described below to evaluate the possible survival benefit. However, ATC is a rare disease and has an extremely short survival period. Moreover, no reliable survival data to refer to have been reported. The meaning of the present findings should thus be evaluated carefully, even though an estimated survival benefit was not observed. The median survival times (MSTs) obtained with a conventional therapeutic strategy and with the study protocol were estimated as 6 and 12 months, respectively. The study entry period was 24 months and the observation period was 12 months, with a type I error (α) level of 0.05 % and type II error (β) level of 0.20. The necessary sample size was calculated as 41. Initially, we set 50 patients as a target size, expecting a 20 % drop-out rate. The protocol was modified in August 2013 to include as many patients as became available until the study period closed, because a higher rate of pathology-based misdiagnoses was reported in another study compared to what we estimated [15]. The overall survival curve will be made by the Kaplan-Meier method.

## Discussion

**Fig. 1:**
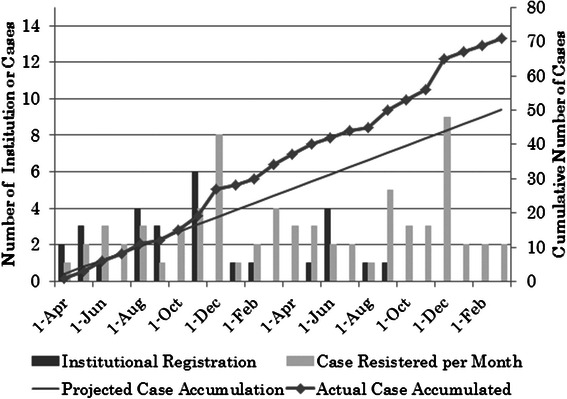
The progress of the present study. Closed bars: Number of institutional registrations (left scale). Open bars: Number of cases registered per month (left scale). Straight line: Projected case accumulation (right scale). Broken line: actual case accumulation (right scale)

The initial proposal of this prospective clinical study was made in 2011 at the annual assembly of the ATCCJ. The initial draft was drawn soon after that by a primary investigator, and it was discussed and refined by a prospective clinical study committee of the ATCCJ. The study was open with the approval of the Ethics Committee of the Osaka City University Medical School in March 2012. The progress of the study is summarized in Fig. 1. The initial patient was enrolled in April 2012, and a total of 71 patients had been enrolled in the study as of the end of March 2014, when the enrollment period was closed. Twenty-eight institutions from around Japan had participated by the end of the enrollment period (Table 1). No severe adverse event to terminate the present study has been reported as of this writing, and an adequate number of patients for an analysis of the feasibility of the protocol was estimated to be enrolled.

The weekly administration of paclitaxel was chosen in the present study because of its high feasibility rate in elderly patients with other malignancies, its widespread use in Japan, its low rate of adverse events compared to other ATC treatments, its ability to be used as an induction therapy, and the capability for concordant irradiation therapy (if needed), in addition to the high efficacy of paclitaxel demonstrated in the initial paper [12]. Many combination therapies have been introduced based on the weekly paclitaxel regimen to treat malignancies of other organs, and future studies of the addition of other anticancer agents to this simple regimen are possible.

During the preparation of this manuscript, the world’s first prospective study targeting patients with ATC was published by Sosa et al. [15]. That study was conducted to examine the additional effect of fosbretabulin in combination chemotherapy with paclitaxel and carboplatin, one of the most common chemotherapeutic regimens for ATC in Western countries [2]. Sosa et al. enrolled 80 patients with mainly stage IVC, and they described the difficulty of enrolling patients based on their distinct eligibility criteria; they noted both the uncertainty in pathological diagnosis and the highly aggressive nature of ATC. They had to exclude as many as 207 patients.

In the present study, our primary endpoints were the feasibility, safety and efficacy of the paclitaxel therapy. Thus, we attempted to enroll as many patients as possible by constructing a system for entry based on the database kept by a nationwide organization (the ATCCJ). Although many patients without a target lesion might have been enrolled, the results of this study were expected to clearly demonstrate the baseline data regarding therapeutic efforts for ATC at present.

A pathological specimen from each patient is being evaluated by the pathology review board of the ATCCJ to confirm the pathological diagnoses of ATC. The primary endpoints of feasibility and efficacy will be evaluated after the expiration of the observation period, in April 2015. Vital information concerning additional treatments after the protocol therapy and the causes of death will be accumulated as well. We hope to analyze the probability of misdiagnoses by pathology, the efficacy of the therapy stratified by clinical staging, and the prognostic index [16].
